# The value of the portable fibrinogen measuring device—a case report of severe postpartum hemorrhage with obstetric disseminated intravascular coagulation

**DOI:** 10.1186/s40981-021-00426-y

**Published:** 2021-03-09

**Authors:** Yoko Hikida, Hiroyuki Sumikura, Hisako Okada, Takashi Fujino, Mayumi Tanaka, Yu Sakai, Shoko Okahara, Rie Inoue

**Affiliations:** 1 Department of Anesthesiology and Pain Medicine, Juntendo University Faculty of Medicine, 3-1-3 Hongo, Bunkyo-ku, Tokyo, 113-8431 Japan; 2 Department of Anesthesiology and Pain Medicine, Juntendo University Graduate School of Medicine, 2-1-1 Hongo, Bunkyo-ku, Tokyo, 113-8431 Japan; 3 Department of Anesthesiology, Yokohama City University, 3-9 Fukuura, Kanazawa-ku, Yokohama, Kanagawa 236-0004 Japan

**Keywords:** Postpartum hemorrhage, Obstetric disseminated intravascular coagulation, Fibrinogen, Point-of-care device, Obstetric anesthesia

## Abstract

**Background:**

Fibrinogen concentration is an important indicator of the treatment for obstetric disseminated intravascular coagulation (DIC). We present how using the fibrinogen measuring device could solve problems in the treatment of postpartum hemorrhage with complicated DIC.

**Case presentation:**

A 32-year-old woman with monochorionic diamniotic twins at 22 weeks of pregnancy was diagnosed with placental abruption and underwent emergent cesarean section. The estimated blood loss was 8375 g. She was transferred to our hospital for further treatment. Compressive uterine sutures and balloon tamponade were performed. We transfused fibrinogen and fresh frozen plasma actively during the operation to maintain plasma fibrinogen above 200 mg/dL by using a point-of-care fibrinogen measuring device. In spite of massive hemorrhage exceeding 10 L, she was extubated at the end of the operation and discharged on the 7th day after the operation.

**Conclusion:**

The portable fibrinogen measuring device was useful for point-of-care assessment of obstetric DIC.

**Supplementary Information:**

The online version contains supplementary material available at 10.1186/s40981-021-00426-y.

## Background

Postpartum hemorrhage (PPH), which rapidly develops acquired hypofibrinogenemia and/or disseminated intravascular coagulation (DIC), is one of the leading causes of maternal mortality. Fibrinogen concentration has been increasingly recognized as an important indicator of the treatment for PPH [1, 2]. Recently, a Japanese manufacturer (A&T Corporation) developed a portable device (CG02N®) that enables point-of-care fibrinogen measurement. We had previously reported a case in which the device was successfully used for the management of massive PPH [3]. In what follows, we present another case where the portable fibrinogen measuring device was useful for the treatment of PPH with more complicated obstetric DIC.

## Case presentation

A 32-year-old woman (157 cm, 47.9 kg, 3 gravida, 2 para) was referred to our hospital due to PPH. She has had a history of 2 previous cesarean deliveries for labor arrest and intrauterine fetal demise. In her third pregnancy, monochorionic diamniotic twins were diagnosed. At 22 weeks of pregnancy, however, the patient was diagnosed with placental abruption and underwent category-1 emergent cesarean section at the previous hospital.

The operation was started under general anesthesia. Her preemie babies were successfully delivered and taken care of by neonatologists. Due to placental abruption, she was suffered from atonic bleeding and coagulopathy. Balloon tamponade with Bakri balloon® was performed before completion of the surgery. However, vaginal bleeding without clot formation continued after surgery in spite of procoagulant supplementation with fresh frozen plasma (FFP). Despite administering 2 mg of recombinant activated factor VII (rFVIIa) and 3000 international units (IU) of freeze-dried concentrated human antithrombin, the estimated blood loss increased to 8375 g. The total amount of transfusion reached to 26 units of packed red blood cells (PRBCs), 30 units of FFP, and 20 units of platelets. The patient was transferred to our hospital for further treatment.

Upon her arrival at our hospital, her blood pressure was 109/89 mmHg, heart rate was 135 bpm (shock index was 1.23), and she was conscious. Continuous bleeding without clotting out of her vagina was found. Along with blood sampling for ordinal laboratory tests, we measured fibrinogen concentration using a portable fibrinogen measuring device at the point of care. It showed 250 mg/dL, with which we judged that her coagulability was tolerable for surgical procedure. Accordingly, we decided to perform another surgery to control her bleeding instead of an arterial embolization by interventional radiology. Laboratory-based test later showed that her plasma fibrinogen level prior to the operation was 243 mg/dL, which matched very well to the result of the portable device (Table 1).

**Table 1:** Time course of fibrinogen concentration and laboratory-based test

		Point-of-care measurement	Laboratory measurement						
	Total amount of bleeding (g)	Fib (mg/dL)	Fib (mg/dL)	Hb (g/dL)	Plt (10^9^/L)	APTT (s)	PT (%)	FDP (μg/mL)	AT (%)
At arrival	8375	250	243	6.2	23	49.7	97	12.7	66
After administration of 3 g of fibrinogen concentration	–	258	–	–	–	–	–	–	–
After administration of 10 U of FFP, 10 U of RBC and 20 U of platelets	10225	261	–	–	–	–	–	–	–
At the end of operation (after additional administration of 2 U of FFP and 2 U of RBC)	10685	256	203	6.4	50	50	90	40.4	57

Along with blood sampling for ordinal laboratory tests, we measured fibrinogen concentration using a portable fibrinogen measuring device at the point of care. A laboratory-based test later showed that her plasma fibrinogen level prior to the operation was 243 mg/dL, which matched very well to the result of the portable device. The laboratory-based test showed low platelets and hemoglobin. We started to transfuse platelets and PRBCs before the operation. Fib fibrinogen, Hb hemoglobin, Plt platelets, APTT activated partial thromboplastin time, PT prothrombin time, FDP fibrin degradation products, AT antithrombin, FFP fresh frozen plasma, PRBCs packed red blood cells

Although her fibrinogen concentration seemed to be an acceptable level for surgery, we administered 3 g of freeze-dried human fibrinogen prophylactically to minimize the risk of bleeding. However, this administration increased the fibrinogen value to 258 mg/dL, which was not as much as we had expected. Therefore, we assessed that her coagulability might be in the hyperfibrinolysis, and decided to transfuse FFP actively during the operation to maintain plasma fibrinogen above 200 mg/dL. We also started to transfuse platelets and PRBCs against low hemoglobin (6.6 g/dL) and platelet counts (23 × 10^9^/L) before the operation (Table 1).

After careful induction of general anesthesia, compressive uterine sutures and balloon tamponade were performed effectively to control her bleeding, and she was extubated at the end of the operation.

As a result, additional blood loss during the surgery was 2310 g, and the amount of transfusion was 12 units of PRBCs, 12 units of FFP, and 20 units of platelets at our hospital. In spite of massive hemorrhage (exceeding 10 L), she was discharged on the 7th day after the operation.

## Discussion

In the current case, the portable fibrinogen measuring device allowed us to provide timely and appropriate treatment for a patient with complicated obstetric DIC. However, some cautions are necessary in handling this device successfully.

There is a glowing consensus that supplementation of fibrinogen is an important component for the treatment of obstetric DIC and that the conventional laboratory-based coagulation tests can be the problem at the time of providing immediate treatment for obstetric DIC because of its time-consuming nature [4, 5]. And some point-of-care devices for fibrinogen measurement have been developed [6]. Our device measures fibrinogen by way of the process of dry-hematology system. It does not require centrifugation and can measure fibrinogen in a whole blood sample within about a minute [7]. Thus, using this device will allow us to provide timely and appropriate management, as it maintains the minimum level of fibrinogen concentration (if not more) necessary for the hemostasis. However, a more thorough and critical examination is required to prove the reliability of the device for the case with lowered fibrinogen concentration such as PPH.

A challenge with providing appropriate treatment for the patient with obstetric DIC is the difficulty of assessing the DIC [8]. Point-of-care viscoelastic testing, such as thromboelastography and thromboelastometry, has been recommended for making a treatment plan for the patient with obstetric DIC [9, 10]. However, substantial skill and experiences are required to correctly evaluate the result [11]. Furthermore, it was still difficult for us to select appropriate treatment based on the analysis of viscoelastic testing, specifically in the current case, where coagulability was modified by various treatments such as rFVIIa or antithrombin. Therefore, we were reluctant to use viscoelastic testing and instead made a treatment plan simply based on plasma fibrinogen value. This successfully resulted in the acceptable amount of bleeding at our hospital. Keeping enough fibrinogen value is a basis of obstetric DIC treatment because fibrinogen is a factor lying in the last stage of coagulation cascade [12, 13]. We think that the management system targeting fibrinogen is especially useful in countries such as Japan, where approximately half of the population goes through clinic-based delivery, and also where there is a shortage of obstetric anesthesiologists. However, other coagulation factors are still needed to form fibrin mesh, and more active supplementation of coagulation factors other than fibrinogen may be required according to the patient’s condition [14], for instance, when bleeding cannot be controlled despite the maintained level of fibrinogen. In the present case, rFVIIa and antithrombin had been already administered at the previous institution, and furthermore, we administered FFP in addition to freeze-dried human fibrinogen. Thus, there is a probability that coagulation factors other than fibrinogen were sufficiently supplemented.

In obstetric DIC, activation of fibrinolysis is common. Since the suppression of hyperfibrinolysis has been suggested as the first step in a therapy algorithm for obstetric DIC, it is important to assess the fibrinolysis. Current evidence suggests that tranexamic acid (TXA) should be used as early as possible for women with established postpartum hemorrhage [15]. However, we did not administrate TXA because it was more than 3 h after bleeding onset [16] and because rFVIIa had been administrated at the previous institution. There have been some reports about embolism related to the administration of rFVIIa in combination with TXA [17, 18]. Therefore, we decided to transfuse FFP actively in order to maintain plasma fibrinogen value without administrating TXA.

In conclusion, the portable fibrinogen measuring device was useful for the management of severe postpartum hemorrhage with complicated obstetric DIC. Further investigation should be made for the effective and enhanced use of the device in the treatment of obstetric DIC.

## Supplementary Information

**Additional file 1.**

## Data Availability

Please contact the author for data requests.

## Abbreviations

PPH: Postpartum hemorrhage
DIC: Disseminated intravascular coagulation
rFVIIa: Recombinant activated factor VII
IU: International units
PRBCs: Packed red blood cells
FFP: Fresh frozen plasma
TXA: Tranexamic acid
